# Nutritional Status of Children Under Five Years in the Slums of West Bengal, India: A Cross-Sectional Study on Prevalence, Characteristics, and Determinants

**DOI:** 10.3390/nu17050853

**Published:** 2025-02-28

**Authors:** Abdul Jaleel, Swapan Bikash Saha, N. Arlappa, Meghendra Banerjee, Samir Narayan Chaudhuri, Mithun Mondal, K. Sreeramakrishna, Ranjith Babu

**Affiliations:** 1 Division of Public Health Nutrition, ICMR-National Institute of Nutrition (NIN), Hyderabad 500007, India; jaleel.cp@icmr.gov.in (A.J.); ramakrishna_nin@yahoo.com (K.S.); 2 Child in Need Institute (CINI), Kolkata 700104, India; swapan@cinindia.org (S.B.S.); meghendra@cinindia.org (M.B.); samir@cinindia.org (S.N.C.); mithun@cinindia.org (M.M.); 3 All India Institute of Medical Sciences (AIIMS), Bibinagar 508126, India; ranjithanthro@gmail.com

**Keywords:** malnutrition, children under five, urbanization, urban slums, India, Kolkata, Siliguri

## Abstract

**Objective:** With rapid urbanization in countries like India, understanding the nutritional status and needs of urban populations, particularly among underprivileged groups such as people living in slums, is crucial. This study investigates the prevalence, characteristics, and determinants of child malnutrition in the urban slums of the Kolkata Municipal Corporation (KMC) and Siliguri Municipal Corporation (SMC) in West Bengal, India. **Methods:** A cross-sectional study was conducted among 736 children aged 6–59 months. Data were collected using structured interviews to gather socioeconomic, demographic, and dietary information, alongside anthropometric measurements. The analysis employed the Composite Index of Anthropometric Failure (CIAF), and multiple linear regression (MLR) models to identify key factors influencing malnutrition. **Results:** The findings revealed a high prevalence of stunting (24.1%), underweight (22.3%), and wasting (15.4%) among children aged 6–59 months, with significant variations observed between the two study sites. Key predictors of anthropometric malnutrition include low household income, incidence of recent illness, low maternal nutrition, and delayed initiation of breastfeeding. **Conclusions:** Addressing child malnutrition in urban slums requires integrated strategies encompassing income-generation opportunities, health-sensitive urban planning, and focused maternal and child health interventions.

## 1. Introduction

Urbanization, a defining feature of the 21st century, has a profound implication for health outcomes on a global scale [1,2,3]. Currently, over 55% of the global population resides in urban areas, a figure projected to reach 68% by 2050 [4,5,6]. This rapid urban expansion, particularly in the Global South [7], raises significant concerns about the health and well-being of economically disadvantaged urban populations. These concerns are underscored by the increasing prevalence of obesity, undernutrition, and micronutrient deficiencies observed in these populations across low- and middle-income countries over the past decade [8].

In many cities, economic disparities force large segments of the population into low-paying, informal employment, limiting their access to adequate housing. As a result, rapid urbanization has fueled the proliferation of slums, particularly in developing regions such as Asia [9]. These slums are typically characterized by substandard housing, overcrowded and poorly planned environments, and inadequate access to sanitation, clean water, and ventilation. Living in such environments perpetuates poverty and exacerbates health inequalities, particularly among vulnerable groups such as children [10].

India has experienced significant urbanization, leading to a rapid increase in slum populations [11]. Currently, an estimated 65.5 million people reside in urban slums across the country [10]. However, this demographic shift has not been accompanied by proportional economic opportunities [12,13], increasing the risk of malnutrition in these settlements. Research indicates that health outcomes of people living in slums are significantly worse than people living in non-slum urban areas [14,15,16,17]. Addressing the health and nutrition needs of this growing urban population will be one of India’s most pressing challenges in the coming decades [18]. Moreover, India’s progress toward Sustainable Development Goal 11—ensuring inclusive, safe, resilient, and sustainable urban development—depends on effectively tackling these issues [19].

Despite the high burden of malnutrition, comprehensive data on children living in slums remain limited. While the National Family Health Survey (NFHS-5) (2019–2021) provides insights into nutritional status of the urban population, it does not specifically assess the determinants of malnutrition in slum settings. At the national level, 30% of children under five in urban areas are stunted, 27% are underweight, and 18.5% are wasted [20]. However, few studies have focused on the nutritional status of children in India’s urban slums [21,22,23], leaving significant knowledge gaps, particularly in densely populated cities beyond the major metropolitan centers.

This study examines the prevalence, characteristics, and determinants of malnutrition among children under five years old in the urban slums of Kolkata Municipal Corporation (KMC) and Siliguri Municipal Corporation (SMC) in West Bengal, India. In Kolkata, where the entire district is urban, 29% of children are stunted, 33% are underweight, and 29% are wasted [10,20]. Moreover, West Bengal has the largest urban population in Eastern India, yet urban malnutrition in this region remains under documented.

By providing a detailed analysis of child malnutrition in these urban slums and identifying potential regional variations, this study aims to inform targeted interventions and guide the development of context-specific nutrition programs. Given that most future urban growth is expected to occur in countries like India, the findings of this research will contribute to improved urban planning and public health strategies, ultimately supporting the well-being of the most disadvantaged populations.

## 2. Materials and Methods

### 2.1. Study Design, Setting, and Population

This cross-sectional study is part of the larger project “Fight Malnutrition and Provide Training to Anganwadi Workers (AWWs) and Nutrition Professionals”, aimed at improving community nutritional outcomes across West Bengal, Assam, and Jharkhand. The current study is part of a larger research project specifically aimed at exploring the facilitators and barriers that influence mothers’ adherence to optimal feeding practices for children aged 0–59 months in urban slums of West Bengal.

Two major cities in West Bengal, Kolkata and Siliguri, were selected as study locations due to their rapid urbanization and the growing challenges posed by expanding slum populations. In Kolkata, the study covered 12 wards under the Kolkata Municipal Corporation (KMC), while in Siliguri, it included 10 wards under the Siliguri Municipal Corporation (SMC). Within each selected ward, two lanes were randomly selected for data collection. The study population consisted of households with at least one child aged 0–59 months. Participation was contingent upon parental consent.

### 2.2. Sample Size

From an initial listing of 3066 households (1491 in KMC and 1575 in SMC), 924 households were identified as eligible, meeting the criterion of having at least one child aged 0–59 months. Of these eligible households, 838 consented to participate in the study (413 in KMC and 425 in SMC). The final analysis focused on children aged 6–59 months for whom complete data on diet and anthropometric measurements were available. This resulted in a final sample of 736 children (362 from KMC and 374 from SMC).

### 2.3. Training and Data Collection

Structured interview schedules were initially developed in English, pre-tested in slum settings, validated by a panel of experts, and subsequently translated into Bengali. Investigators selected for data collection underwent five days of comprehensive training. The training included detailed instructions on administering the pre-tested interview schedules, which were designed to gather socioeconomic and demographic information about households and document the history of morbidity among children in the preceding two weeks. Additionally, investigators received training on dietary data collection, focusing on recording foods and liquids consumed by the child in the previous 24 h using a structured, list-based interview schedule [21,22]. Field staff were also trained to measure the height/length and weight of children and mothers using standardized equipment and established protocols [23]. To ensure accuracy, investigators were standardized to minimize inter- and intra-individual variations in anthropometric measurements.

### 2.4. Anthropometry

The weight of each child and mother was measured using a Seca 813 digital scale (Seca, Hamburg, Germany), with readings recorded to the nearest 0.01 kg. For children under one year of age or those unable to stand independently on the scale, weight was determined by subtracting the caregiver’s weight from the combined weight of the caregiver holding the child. Both caregivers and children were measured in light clothing and without shoes or footwear. The height of mothers and children over two years of age was measured using a Seca 213 stadiometer, with measurements taken to the nearest 0.1 cm. For children under two years of age or those shorter than 85 cm, recumbent length was measured using a Seca 417 infantometer, also recorded to the nearest 0.1 cm.

### 2.5. Outcome Variables and Definitions

The outcome variables assessed in this study included stunting, underweight, wasting, and overweight. Z-scores for height-for-age (HAZ), weight-for-age (WAZ), and weight-for-height (WHZ) were computed using the WHO Anthro software (version 3.2 2), based on each child’s date of birth, height/length (in cm), and weight (in kg) [24]. Stunting was defined as a height-for-age Z-score (HAZ) below −2 standard deviations (SDs). Underweight was classified as a weight-for-age Z-score (WAZ) below −2 SDs, while wasting was defined as a weight-for-height Z-score (WHZ) below −2 SDs. Overweight was determined as a WHZ above +2 SDs.

### 2.6. Ethical Consideration

This study received approval from the Institutional Ethical Committee (IEC) of the Child in Need Institute (CINI), Kolkata, under reference number IEC-03/2-23-24 on 01-11-2022. All investigators underwent comprehensive training on the study’s ethical protocols. During data collection, the research team strictly adhered to ethical principles to safeguard the dignity, rights, and welfare of all participants. Informed consent was obtained from both the head of the household and the child’s mother prior to data collection. Participants were fully informed about the voluntary nature of their participation and assured that all data collected would be handled with strict confidentiality. The findings of this study will be shared with key stakeholders, including relevant government departments.

### 2.7. Statistical Analysis

Data analysis was conducted using Stata software (version Stata-14). The normality of continuous variables (HAZ, WAZ, and WHZ) was assessed using the Shapiro–Wilk test, which confirmed a normal distribution (*p* > 0.05). The prevalence of malnutrition and various socioeconomic and demographic characteristics were reported with 95% confidence intervals (CIs). To provide a comprehensive understanding of anthropometric malnutrition, the Composite Index of Anthropometric Failure (CIAF) was calculated [25]. This index captures all nine biologically plausible forms of anthropometric malnutrition among children under five years of age. The determinants of malnutrition were analyzed using multiple linear regression (MLR), with WHZ, HAZ, and WAZ as the dependent variables. A total of 27 potential covariates (Supplementary File, Table S1) were selected based on the UNICEF Conceptual Framework on the Determinants of Maternal and Child Nutrition—2020 [26]. Multi-collinearity among independent variables was evaluated using the variance inflation factor (VIF) test. All VIF values were below 10, and tolerance values exceeded 0.2, indicating no significant multi-collinearity (Supplementary File, Table S1). The MLR model was subsequently applied, and the coefficient of determination (R^2^) exceeded 0.5, demonstrating that the model provided a reasonably good fit for the data. Only variables with statistically significant *p*-values (*p* < 0.05) were included in the final MLR table.

## 3. Results

### 3.1. Characteristics of the Study Population

Table 1 summarizes the demographic, nutritional, social, and health characteristics of the study population. The average age of children was 31 months in KMC slum areas and 29 months in SMC slum areas. Children aged 6–23 months comprised 36% of the sample in KMC and 41.4% in SMC. The average maternal age was 27 years in KMC and 25.5 years in SMC. More than 40% of mothers in both areas were classified as having short stature (height < 150 cm). Educational attainment was generally low, with over half of the mothers (52% in KMC and 56% in SMC) and a significant proportion of fathers (56% in KMC and 66% in SMC) not completing the eighth grade.

### 3.2. Short-Term Morbidities Among Children

In the two weeks preceding the survey, 61% of children in KMC slums and 58% in SMC slums exhibited symptoms of respiratory illness, including a cough or runny nose. Fever was reported in 27% of children in KMC and 17% in SMC. Additionally, the prevalence of diarrhea was 7.1% in KMC and 3.2% in SMC during the same period.

### 3.3. Malnutrition Among Children

The mean birth weight across both study sites was 2.8 kg. However, low birth weight (<2.5 kg) was common, affecting 21% of children in KMC and 25% in SMC. Among children aged 6–59 months, stunting prevalence was 17.7% in KMC and 30.4% in SMC. Wasting was higher in KMC (17%) than in SMC (13.7%). Severe acute malnutrition (SAM) affected 6% of children in KMC and 5% in SMC. Additionally, approximately 20% of children in both areas were classified as underweight.

**Figure 1:**
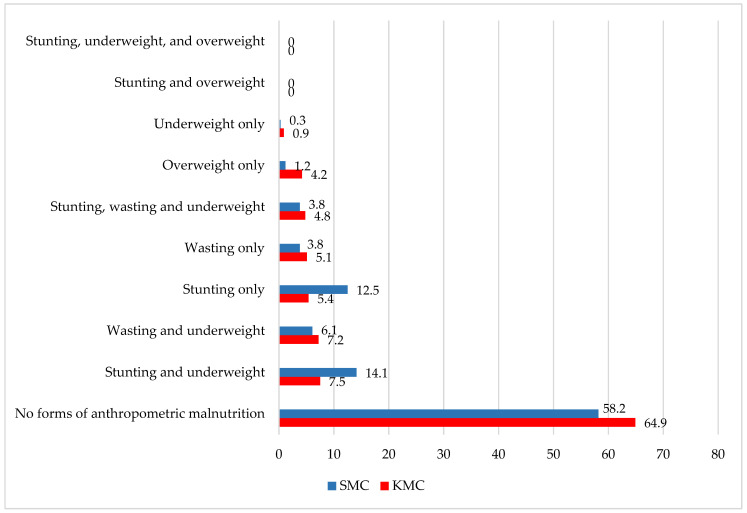
Prevalence of different forms of anthropometric malnutrition (%) among children (6–59 months) in the study locations.

Figure 1 presents the distribution of anthropometric failure among children in the slums of KMC and SMC. This was assessed using the Composite Index of Anthropometric Failure (CIAF), which provides a comprehensive measure beyond traditional indicators like underweight, stunting, and wasting. The CIAF captures all possible forms of anthropometric failure, helping to identify the most vulnerable children and their specific nutritional needs. The analysis showed that 35.1% of children in KMC and 41.8% in SMC experienced at least one form of anthropometric failure. In KMC, the most common forms were the coexistence of stunting and underweight (7.5%) and wasting and underweight (7.2%). In SMC, the most prevalent was stunting and underweight (14.1%), followed by stunting alone (12.5%) and wasting and underweight (6.1%). Additionally, overweight was observed in 4.2% of children in KMC, reflecting the dual burden of malnutrition in urban slum settings.

### 3.4. Predictors of Poor Growth Among Children 6–59 Months

#### 3.4.1. Height-for-Age Z-Score

The analysis identified several key predictors of lower HAZ scores (Table 2). Older children, those who experienced delayed breastfeeding initiation (after one hour of birth), and those living in kutcha houses were more likely to have lower HAZ scores. Age showed a negative association with HAZ, with the lowest scores observed among children aged 24–35 months and 36–47 months in KMC. In SMC, the lowest scores were observed among those aged 12–23 months and 36–47 months. While delayed breastfeeding initiation had a marginal negative association with HAZ in the overall sample, it was significantly linked to lower HAZ scores in KMC (coefficient = −0.626, *p* = 0.017). Similarly, residing in a kutcha house was significantly associated with lower HAZ scores in the overall sample (coefficient = −0.555, *p* = 0.030). In contrast, children from households with more than four members (only in KMC), those with higher monthly incomes (above INR 5000) (in the overall sample and in KMC), and those with mothers taller than 150 cm (in both KMC and SMC) exhibited significantly higher HAZ scores.

#### 3.4.2. Weight-for-Height Z-Score

The analysis identified age, recent short-term morbidities, household income, and maternal BMI as significant predictors of lower WHZ scores (Table 2). Compared to children aged 6–11 months, those aged 12–23 months had significantly higher WHZ scores. This increase was statistically significant in the overall sample (coefficient = 0.600, *p* = 0.003) and marginally significant in the KMC sample (coefficient = 0.616, *p* = 0.063). Children who had not experienced any illness in the previous 15 days had higher WHZ scores than those who had, suggesting that recent illnesses significantly contribute to wasting in slum settings. Children from households’ income more than INR 20,000 per month had significantly higher WHZ scores (coefficient = 1.035, *p* < 0.001 in the overall sample and coefficient = 1.299, *p* = 0.002 in KMC). However, in the SMC area, no significant association was found between income levels and WHZ scores. Children of mothers with a BMI ≥ 30 kg/m^2^ had significantly higher WHZ scores. Similarly, positive associations were found for mothers with BMIs in the 18.5–24.9 kg/m^2^ and 25–29.9 kg/m^2^ ranges in both the overall and SMC samples, though these associations were not statistically significant in the KMC sample.

#### 3.4.3. Weight-for-Age Z-Score

Household income, maternal height, recent morbidities, and Caste/Tribe emerged as significant predictors of low weight-for-age (Table 2). Higher household income was associated with better WAZ scores, with significant improvements observed at monthly incomes of INR 5000 and above. Maternal height (≥150 cm) was also significantly linked to higher WAZ scores. In Kolkata, both maternal height and higher maternal BMI were positively associated with WAZ scores. However, in Siliguri, maternal height remained a significant predictor of improved WAZ scores, while maternal BMI showed a weaker, non-significant association. Children who had not experienced morbidity in the past 15 days had significantly higher WAZ scores than those who had (coefficient = 0.302, *p* = 0.007). Caste and tribal status also played a role; children from Scheduled Tribes (ST) had significantly higher WAZ scores than those from Scheduled Castes (SC) in both the overall sample (coefficient = 0.290, *t* = 2.06, *p* = 0.040) and in Siliguri (coefficient = 0.481, *t* = 2.82, *p* = 0.005).

**Table 2:** Standardized coefficients with *p*-values from the multiple linear regression analysis.

	Overall			KMC			SMC		
	Coef.	t	*p* Value	Coef.	t	*p* Value	Coef.	t	*p* Value
**Height-for-age Z-score**									
**Age of the child**									
6–11 months	1			1			1		
12–23 months	−0.535	−2.38	0.018	−0.287	−0.87	0.385	−0.875	−2.59	0.010
24–35 months	−1.059	−4.2	0.000	−0.731	−2.05	0.042	−1.201	−3.11	0.002
36–47 months	−0.835	−3.4	0.001	−0.707	−2.06	0.040	−1.222	−3.13	0.002
48–59 months	−0.860	−3.37	0.001	−0.757	−2.1	0.037	−0.968	−2.42	0.016
**Initiation of breastfeeding**									
Within 1 h of birth	1			1			1		
Witin 1 to 2 h of birth	−0.319	−1.67	0.096	−0.626	−2.41	0.017	−0.025	−0.08	0.935
After 2 h and within 24 h	−0.091	−0.49	0.622	−0.256	−0.94	0.346	0.138	0.51	0.613
**Type of house**									
Pucca	1			1			1		
Semi-Pucca	−0.179	−1.11	0.267	−0.019	−0.08	0.939	−0.178	−0.74	0.463
Kutcha	−0.555	−2.18	0.030	−0.376	−1.02	0.308	−0.329	−0.81	0.419
**Household size**									
<4 members	1			1			1		
4–6 members	0.390	1.9	0.057	0.653	2.01	0.046	0.010	0.03	0.974
More than 7 members	0.245	1.07	0.286	0.292	0.79	0.430	0.259	0.85	0.399
**Monthly income**									
<INR 5000	1			1			1		
INR 5000 to 9999	0.781	2.59	0.010	1.294	3.39	0.001	0.050	0.08	0.934
INR 10,000 to 14,999	1.043	3.48	0.001	1.004	2.7	0.008	0.576	0.97	0.334
INR 15,000 to 19,999	1.044	3.25	0.001	1.282	3.08	0.002	0.521	0.86	0.391
More than INR 20,000	0.994	3.19	0.001	1.156	2.83	0.005	0.414	0.7	0.485
**Maternal height**									
<150 cm	1			1			1		
≥150 cm	0.602	4.83	0.000	0.762	4.11	0.000	0.574	3.21	0.002
**Weight-for-height Z-score**									
**Age of the child**									
6–11 months	1			1			1		
12–23 months	0.600	2.98	0.003	0.616	1.87	0.063	0.231	0.8	0.425
24–35 months	0.699	3.1	0.002	0.838	2.35	0.020	0.262	0.79	0.428
36–47 months	0.815	3.71	0.000	1.001	2.92	0.004	0.282	0.85	0.399
48–59 months	0.668	2.92	0.004	0.823	2.28	0.023	0.257	0.75	0.455
**Any morbidity in last 15 days**									
No	1			1			1		
Yes	0.317	2.72	0.007	0.433	2.2	0.029	0.217	1.35	0.179
**Monthly income**									
<INR 5000	1			1			1		
INR 5000 to 9999	0.830	3.07	0.002	0.988	2.59	0.010	0.257	0.5	0.614
INR 10,000 to 14,999	0.717	2.67	0.008	0.984	2.64	0.009	0.002	0	0.997
INR 15,000 to 19,999	0.733	2.55	0.011	0.970	2.34	0.020	0.225	0.43	0.665
More than INR 20,000	1.035	3.71	0.000	1.299	3.19	0.002	0.478	0.95	0.345
**Maternal BMI**									
<18.5 kg/m^2^	1			1			1		
18.5 to 24.9 kg/m^2^	0.329	1.9	0.058	0.391	1.37	0.173	0.491	2.1	0.037
15 to 29.9 kg/m^2^	0.494	2.57	0.010	0.525	1.69	0.092	0.623	2.29	0.023
≥30 kg/m^2^	0.908	3.84	0.000	0.955	2.64	0.009	0.711	2	0.047
**Weight-for-age Z-score**									
**Any morbidity in last 15 days**									
No	1			1			1		
Yes	0.302	2.72	0.007	0.227	1.24	0.218	0.247	1.64	0.103
**Vit A supplementation**									
Received	1			1			1		
Not received	0.122	1.06	0.291	0.399	2.15	0.033	−0.053	−0.32	0.749
**Caste/Tribe**									
Scheduled Caste (SC)	1			1			1		
Scheduled Tribes (ST)	0.290	2.06	0.040	−0.011	−0.04	0.967	0.481	2.82	0.005
Other Backward Classes (OBCs)	−0.315	−1.39	0.164	0.124	0.26	0.793	−0.171	−0.63	0.532
Others (Other than SC/ST/OBC)	0.159	0.88	0.382	0.372	1.32	0.187	0.175	0.66	0.507
**Monthly income**									
< INR 5000	1			1			1		
INR 5000 to 9999	1.032	4.00	0.000	1.405	3.94	0.000	0.220	0.46	0.645
INR 10,000 to 14,999	1.106	4.32	0.000	1.264	3.64	0.000	0.342	0.72	0.474
INR 15,000 to 19,999	1.099	4.01	0.000	1.374	3.55	0.000	0.459	0.95	0.345
More than INR 20,000	1.283	4.82	0.000	1.516	3.98	0.000	0.583	1.23	0.220
**Maternal height**									
<150 cm	1			1			1		
≥150 cm	0.431	4.05	0.000	0.502	2.9	0.004	0.439	3.06	0.002
**Maternal BMI**									
<18.5 kg/m^2^	1			1			1		
18.5 to 24.9 kg/m^2^	0.287	1.74	0.083	0.348	1.3	0.194	0.394	1.8	0.073
15 to 29.9 kg/m^2^	0.381	2.08	0.038	0.418	1.44	0.150	0.436	1.71	0.089
≥30 kg/m^2^	0.839	3.72	0.000	0.809	2.4	0.017	0.608	1.82	0.070

## 4. Discussion

### 4.1. Status of Childhood Malnutrition in Urban Slums of West Bengal

This study provides an assessment of the nutritional status of children living in urban slums in West Bengal, India. The findings indicate that the overall prevalence of stunting, wasting, and underweight was 24.1%, 15.4%, and 22.3%, respectively. Notably, stunting and underweight were more prevalent in Siliguri Municipal Corporation (SMC); while wasting and overweight were more common in Kolkata Municipal Corporation (KMC). Additionally, 5% of children exhibited severe growth faltering, experiencing the co-occurrence of stunting, wasting, and underweight, a condition associated with a significantly increased risk of mortality [27]. Compared to previous studies conducted in urban slums across Maharashtra, Mumbai, Bhubaneswar, and Hyderabad, the prevalence of malnutrition in our study was relatively lower [28,29,30,31]. However, the prevalence of wasting is significantly higher in the slums of KMC.

One possible explanation for the lower overall prevalence of malnutrition observed in this study is the selection of older slum settlements, which tend to have better-developed health infrastructure and improved access to basic services. Research suggests that old slums in larger Indian cities generally have better access to public water, private toilets, and domestic electricity connections compared to those in smaller cities and urban peripheries [11]. A recent study investigating water, sanitation, and hygiene (WASH) infrastructure in urban slums of Kolkata also revealed relatively favorable conditions that over 90% of households had piped water access, nearly universal access to improved latrines, and high adherence to food hygiene practices [32].

However, conditions may be markedly different in newly emerging urban slums, particularly in peri-urban areas settled by recent migrants or individuals displaced to the urban fringes due to high living costs in central locations. Studies indicate that peri-urban areas of Kolkata are undergoing rapid population growth, whereas the urban core is experiencing stagnant or negative growth [33]. Future research should therefore expand beyond established slums to include peri-urban regions and rural–urban transition zones to gain a more holistic understanding of urban malnutrition.

### 4.2. Short-Term Morbidities, Early Childhood Wasting, and Later Growth Faltering

A significant finding of this study was the high prevalence of short-term morbidities, affecting 61% of children in KMC and 58% in SMC. This study also found that children who had not experienced illness in the preceding 15 days had significantly higher WHZ and WAZ scores, emphasizing the importance of reducing disease exposure. Recurrent infections have a well-documented association with wasting [34], and early-childhood wasting has been linked to subsequent growth faltering, including concurrent wasting and stunting, which significantly increases the risk of mortality [35].

In this study, wasting was found to be most prevalent among children aged 0–2 years, however children over 24 months were more likely to be stunted. Prolonged exposure to recurrent infections, early wasting, poor dietary practices, and inadequate caregiving environments are likely contributors to this trend. Similar patterns have been reported in other studies. For instance, a cohort study in Bangladesh found that stunting prevalence increased from 18% at birth to 48% by 24 months [36], and global analyses indicate that stunting peaks around 28 months [37,38].

Given that wasting is most prevalent among children aged 0–2 years, interventions must be prioritized to mitigate its long-term consequences, including its role in later stunting [39]. Addressing environmental factors that contribute to frequent childhood illnesses is equally important. Unsafe drinking water, poor sanitation, inadequate waste management, and substandard housing conditions increase the risk of infections that lead to early wasting.

### 4.3. Child Malnutrition and Maternal Factors

Maternal nutrition plays a critical role in preventing childhood malnutrition. Consistent with previous findings, this study identified maternal height (≥150 cm) as a predictor of stunting and underweight [40]. Furthermore, we also found that children of mothers with higher BMIs were less likely to suffer from wasting. Research suggests that improving maternal nutritional status during adolescence and pregnancy positively influences children’s linear growth [41]. Additionally, delayed initiation of breastfeeding is also a significant predictor of stunting, particularly in the slums of Kolkata. Early breastfeeding initiation is widely recognized as a protective factor against stunting [42], infant morbidity [43], and neonatal and early infant mortality [44,45]. These findings align with previous research linking wasting to younger age, delayed breastfeeding initiation, diarrheal illnesses, poor dietary diversity, and low socioeconomic status [46,47,48].

### 4.4. Child Malnutrition and Household Factors

Larger households (4–6 members) were significantly associated with higher HAZ scores in Kolkata. This may be due to the shared caregiving responsibilities within extended families, which can positively impact child development [49,50]. The presence of additional caregivers may improve prenatal and postnatal care, child supervision, feeding practices, and overall well-being during critical developmental years. Higher household income was also significantly associated with better nutritional outcomes (higher HAZ and WHZ scores). This finding is consistent with studies demonstrating that improved financial resources lead to better access to quality food, healthcare, and living conditions, ultimately reducing stunting and wasting [51,52]. Increased financial resources allow families to access better-quality food, healthcare, and improved living conditions, all of which contribute to enhanced child growth outcomes. However, in urban slum settings, households in the poorest areas often spend up to 75% of their income on food [53], leaving little financial flexibility for healthcare or other essential needs. These findings highlight the need for economic interventions aimed at increasing household income to combat malnutrition in vulnerable urban populations.

However, this study has limitations. Its focus on only two urban centers may limit the generalizability of findings to all slums in West Bengal or eastern India. Additionally, its cross-sectional design restricts causal interpretations. Future research should adopt a longitudinal approach and incorporate peri-urban areas and newly emerging slums to gain a more comprehensive understanding of urban malnutrition. Mixed-method and qualitative studies are also necessary to explore the socioeconomic and behavioral drivers of malnutrition among people living in urban slum.

## 5. Conclusions

This study highlights the significant burden of stunting, underweight, wasting, and short-term morbidities among children under five living in the urban slums of Kolkata and Siliguri. The findings indicate that child malnutrition is influenced by multiple factors, including age, household income, incidence of short-term morbidities, maternal nutritional status (height and BMI), and delayed initiation of breastfeeding. Addressing malnutrition in urban slums requires comprehensive, multi-sectoral interventions. Efforts should focus not only on improving healthcare and nutrition services but also on tackling the broader determinants of poverty through policies that promote income-generating opportunities for disadvantaged urban populations. Additionally, health-sensitive urban planning is essential to creating environments that support better nutrition and overall well-being. Strengthening government–NGO partnerships could facilitate targeted interventions, such as housing improvements, nutrition education, and community-based health programs, to effectively reduce the burden of malnutrition and improve the health outcomes of vulnerable children in urban slums.

## Figures and Tables

**Table 1:** Characteristics of the children (6–59 months) and their mothers included in the study.

Characteristics	KMC		SMC		Overall	
	*n*	Mean or Percent [95% CI]	*n*	Mean or Percent [95% CI]	*n*	Mean or Percent [95% CI]
Child’s age (mo) (mean)	362	31.0 [29.5–32.5]	374	29.0 [27.5–30.5]	736	30.0 [28.9–31.1]
Age (6–23 mo) (%)	362	36.2 [31.4–41.3]	374	41.4 [36.5–46.5]	736	38.8 [35.4–42.4]
Birth weight (kg) (mean)	295	2.8 [2.7–2.9]	328	2.8 [2.7–2.8]	623	2.8 [2.7–2.8]
Low birthweight (<2.5 kg) (%)	295	20.7 [16.4–25.7]	328	24.7 [20.3–29.7]	623	22.8 [19.7–26.3]
Weight (kg) (mean)	342	12.2 [11.5–12.8]	353	11.6 [10.8–12.5]	695	11.9 [11.4–12.4]
Length/height (cm) (mean)	341	88.0 [86.8–89.2]	352	84.8 [83.7–85.9]	693	86.4 [85.5–87.2]
Sex of the child: Female (%)	362	49.4 [44.3–54.6]	374	49.5 [44.4–54.5]	736	49.5 [45.8–53.1]
Stunting (HAZ < −2 SD) * (%)	334	17.7 [13.9–22.2]	342	30.4 [25.7–35.5]	676	24.1 [21.0–27.5]
Severe stunting (HAZ < −3 SD) * (%)	334	6.6 [4.3–9.8]	342	9.0 [6.4–12.6]	676	7.8 [6.0–10.1]
Height-for-age Z-score (mean)	334	−0.7 [−0.8–−0.5]	342	−1.1 [−1.3–−1.0]	676	−0.9 [−1.0–−0.8]
Wasting (WHZ < −2 SD) * (%)	334	17.1 [13.4–21.5]	342	13.7 [10.5–17.8]	676	15.4 [12.8–18.3]
Severe wasting (WHZ < −3 SD) * (%)	334	6.3 [4.1–9.5]	342	4.9 [3.1–7.8]	676	5.6 [4.1–7.6]
Weight-for-height Z—score (mean)	334	−0.7 [−0.9–−0.6]	342	[−0.9–−0.6]	676	−0.7 [−0.8–−0.6]
Underweight (WAZ < −2 SD) * (%)	334	20.3 [16.3–25.0]	342	24.3 [20.0–29.1]	676	22.3 [19.3–25.6]
Severe underweight (WAZ < −3 SD) * (%)	334	6.6 [4.3–9.8]	342	4.7 [2.8–7.5]	676	5.6 [4.1–7.6]
Weight-for-age Z- score (mean)	334	−0.9 [−1.1–−0.8]	342	−1.2 [−1.3–−1.0]	676	−1.0 [−1.1–−0.9]
Diarrhea within past 15 days (%)	362	7.1 [4.9–10.3]	374	3.2 [1.8–5.6]	736	5.1 [3.8–7.0]
Fever within past 15 days (%)	362	27.3 [23.0–32.2]	374	16.6 [13.1–20.7]	736	21.8 [19.0–25.0]
Cough/running nose within past 15 days (%)	362	60.7 [55.6–65.7]	374	58.3 [53.2–63.2]	736	59.5 [55.9–63.0]
Maternal age (years) (mean)	362	27.0 [26.5–27.5]	374	25.5 [25.0–26.0]	736	26.2 [25.9–26.6]
Maternal height (cm) (mean)	336	151.3 [150.6–151.9]	354	150.5 [149.9–151.1]	690	150.9 [150.4–151.3]
Maternal short stature (height < 150 cm) (%)	336	40.8 [35.6–46.1]	354	42.1 [37.0–47.3]	690	41.4 [37.8–45.2]
Maternal education ≤ 8 standard (%)	362	52.2 [47.0–57.3]	374	55.6 [50.5–60.6]	736	53.9 [50.3–57.5]
Paternal education ≤ 8 standard (%)	362	55.5 [50.3–60.6]	374	65.5 [60.5–70.2]	736	60.6 [57.0–64.0]

* The WHO classifies children under five as undernourished based on their growth measurements compared to the WHO Child Growth Standards. Undernutrition is assessed using three key indicators: Stunting—assessed by height-for-age (HAZ): stunted—HAZ < −2 standard deviations from the median; severely stunted—HAZ < −3 SD from the median. Wasting—assessed by weight-for-height (WHZ): wasted—WHZ < −2 SD from the median; severely wasted—WHZ < −3 SD from the median. Underweight—assessed by weight-for-age (WAZ): underweight—WAZ < −2 SD from the median; severely underweight—WAZ < −3 SD from the median.

## Data Availability

The data will be available as per request. The data are not publicly available due to privacy.

## Supplementary Materials

The following supporting information can be downloaded at: https://www.mdpi.com/article/10.3390/nu17050853/s1, Table S1: List of independent variables included in the model and collinearity statistics.
