# Supplementary material for: Nutritional Status of Children Under Five Years in the Slums of West Bengal, India: A Cross-Sectional Study on Prevalence, Characteristics, and Determinants
Source: Nutrients. 2025 Feb 28;17(5):853. doi: 10.3390/nu17050853 (PMC11902011; doi:10.3390/nu17050853)
Supplement: Supplementary file 1 [file nutrients-17-00853-s001.zip › nutrients-3471749-supplementary.pdf]

Table S1. List of independent variables included in the model and collinearity statistics

**Input variables**	**VIF**	**1/VIF**
Child's age	1.26	0.794
Sex of the child	1.05	0.952
Any morbidity	1.08	0.923
IFA syrup received	1.2	0.831
Vita A supplementation received	1.18	0.845
Deworming tablet/syrup received	1.24	0.804
ICDS service received	1.15	0.868
Initiation of breastfeeding	1.26	0.791
Feeding of colostrum	1.21	0.823
Fed anything before initiating breastfeeding	1.14	0.874
Religion	1.22	0.820
Caste/Tribe	1.05	0.951
Length of stay in the current locality	1.17	0.851
Type of house	1.53	0.653
Household size	1.1	0.908
Monthly income	1.24	0.808
Cooking fuel using	1.52	0.660
Source of drinking water	1.23	0.814
Toilet facility available	1.27	0.788
Maternal education	1.46	0.685
Paternal education	1.43	0.698
Mather's occupation	1.17	0.854
Father's occupation	1.28	0.781
Maternal age	1.11	0.901
Maternal height	1.05	0.953
Maternal BMI	1.14	0.879
Diet Diversity (children)	1.12	0.891
